# Memory formation for trace fear conditioning requires ubiquitin-proteasome mediated protein degradation in the prefrontal cortex

**DOI:** 10.3389/fnbeh.2013.00150

**Published:** 2013-10-23

**Authors:** David S. Reis, Timothy J. Jarome, Fred J. Helmstetter

**Affiliations:** Department of Psychology, University of Wisconsin-MilwaukeeMilwaukee, WI, USA

**Keywords:** memory, ubiquitin, protein degradation, protein synthesis inhibitors, fear conditioning, trace conditioning, prefrontal cortex

## Abstract

The cellular mechanisms supporting plasticity during memory consolidation have been a subject of considerable interest. *De novo* protein and mRNA synthesis in several brain areas are critical, and more recently protein degradation, mediated by the ubiquitin-proteasome system (UPS), has been shown to be important. Previous work clearly establishes a relationship between protein synthesis and protein degradation in the amygdala, but it is unclear whether cortical mechanisms of memory consolidation are similar to those in the amygdala. Recent work demonstrating a critical role for prefrontal cortex (PFC) in the acquisition and consolidation of fear memory allows us to address this question. Here we use a PFC-dependent fear conditioning protocol to determine whether UPS mediated protein degradation is necessary for memory consolidation in PFC. Groups of rats were trained with auditory delay or trace fear conditioning and sacrificed 60 min after training. PFC tissue was then analyzed to quantify the amount of polyubiquibated protein. Other animals were trained with similar procedures but were infused with either a proteasome inhibitor (clasto-lactacystin β-lactone) or a translation inhibitor (anisomycin) in the PFC immediately after training. Our results show increased UPS-mediated protein degradation in the PFC following trace but not delay fear conditioning. Additionally, post-training proteasome or translation inhibition significantly impaired trace but not delay fear memory when tested the next day. Our results further support the idea that the PFC is critical for trace but not delay fear conditioning and highlight the role of UPS-mediated degradation as critical for synaptic plasticity.

## Introduction

Pavlovian fear conditioning has proven to be exceptionally useful in elucidating the molecular mechanisms underlying learning and memory. This procedure involves the association of a conditional stimulus (CS) with an aversive unconditional stimulus (UCS). Through repeated pairings of these two stimuli the CS becomes a predictor of the UCS and the subject will emit a fear response to the CS alone. In the most commonly used version of Pavlovian fear conditioning, “delay” fear conditioning, the UCS normally occurs at CS offset. The acquisition and storage of this association requires the amygdala (Fanselow and LeDoux, 1999; Wilensky et al., 2006; Helmstetter et al., 2008; Pape and Pare, 2010). Several studies have shown that the consolidation of fear memory depends on mRNA transcription and translation of new protein in the amygdala and that inhibiting these processes prevents the formation of a stable fear memory (Bailey et al., 1999; Parsons et al., 2006; Helmstetter et al., 2008; Kwapis et al., 2011). In addition, protein degradation, mediated by the ubiquitin-proteasome system (UPS), is another critical regulatory mechanism in synaptic plasticity required for memory (Hegde et al., 1997; Jarome et al., 2011). Protein degradation triggered by neural activity may be a key factor in making synapses labile, which is crucial for both memory consolidation and “reconsolidation” (Jarome and Helmstetter, 2013).

Proteins are marked for degradation through the covalent attachment of ubiquitin tags. The ubiquitin proteins are attached through the action of an enzymatic pathway consisting of 3 enzymes, termed E1, E2, and E3 (Hershko and Ciechanover, 1998). This pathway is able to add additional ubiquitin molecules to an already substrate-bound ubiquitin at different lysine (K) residues, thus creating a polyubiquitin chain. These chains act as molecular signals for a variety of cellular processes, depending upon the lysine residue at which they are linked (Deng et al., 2000; Ye and Rape, 2009). Polyubiquitin chains linked together at the lysine-48 (K48) residue of ubiquitin are likely to be degradation specific (Hegde, 2010). Proteins tagged with K48 polyubiquitin chains are targeted by the 26S proteasome and subsequently degraded.

Ubiquitin-proteasome mediated protein degradation is critical for memory consolidation and reconsolidation in several forms of learning. For example, an infusion of the proteasome inhibitor, clasto-lactacystin- β-lactone (β-lac) into the CA1 region of hippocampus after retrieval prevents anisomycin-induced memory deficits and extinction of a context fear memory (Lee et al., 2008). Infusion of a different proteasome inhibitor (lactacystin) into the CA3 region of the hippocampus impairs the consolidation and reconsolidation of a spatial memory (Artinian et al., 2008). Recently, Jarome et al. (2011) showed that the consolidation of fear conditioning requires UPS-mediated protein degradation in the amygdala. Post-training infusions of β-lac into the amygdala of rats immediately following training in delay fear conditioning (DFC) impaired the formation of conditional responses. Thus, UPS-mediated protein degradation may represent a common mechanism supporting synaptic plasticity and memory consolidation in multiple brain areas. As mentioned above, *de novo* protein synthesis is critical for the formation of trace fear memory in the amygdala (Kwapis et al., 2011) but there have been very few studies that have investigated a similar role for protein synthesis in the prefrontal cortex. One study infused the protein synthesis inhibitor anisomycin into the PFC of rats immediately following trace fear training. When tested for fear to the CS 30 days later, it was found that post-training inhibition of protein synthesis impaired memory (Blum et al., 2006). However, since this study only addressed the role of protein synthesis on memory tested remotely, it remains unknown whether protein synthesis in the PFC is necessary for initial consolidation of trace fear memory. Furthermore, no study has investigated the role of UPS-mediated protein degradation in the formation and consolidation of trace fear memory.

Despite a wealth of information regarding the mechanisms underlying delay fear memory, much less is known about those supporting the consolidation of memory for more complex variations of Pavlovian fear conditioning, such as trace fear conditioning. Unlike DFC, the CS and UCS in trace fear conditioning are not temporally contiguous. Instead, they are separated by a brief stimulus free interval during training. Associating the CS and UCS across this trace interval requires structures in addition to the hippocampus and amygdala (McEchron et al., 1998; Esclassan et al., 2009; Gilmartin and Helmstetter, 2010; Czerniawski et al., 2011; Guimarais et al., 2011; Kwapis et al., 2011; Gilmartin et al., 2012). One structure that has gained significant attention in this regard is the prefrontal cortex (PFC). Importantly, the PFC has been shown to be necessary for auditory trace but not DFC. Gilmartin and Helmstetter (2010) demonstrated that inactivation of the prelimbic region of PFC (PL), as well as the blockade of NMDAR-mediated synaptic transmission in PFC, significantly attenuated the acquisition of fear to a trace CS further supporting the importance of the PFC to trace but not DFC. Additionally, the PFC is involved in the long-term storage of trace fear memories suggesting that the PFC is necessary not only for the acquisition of trace fear memory but also for the storage of trace fear memory (Runyan et al., 2004).

Our study focused on the role of UPS-mediated protein degradation and protein synthesis in the PFC following trace vs. DFC in rats. Specifically, we examined if (1) degradation specific polyubiquitin tagging was selectively increased following trace but not DFC and if (2) post-training inhibition of the 26S proteasome or *de novo* protein synthesis in the PFC impaired the consolidation of trace but not delay fear memory.

## Materials and methods

### Animals and surgery

The experiments used 87 male Long Evans rats (~300–400 g; Harlan, Madison, WI). The rats were individually housed with *ad libitum* access to food and water. The colony room was maintained under a 14:10-h light/dark cycle, and all behavioral tests were conducted during the light portion of the cycle. All procedures were approved by the Institutional Animal Care and Use Committee (University of Wisconsin-Milwaukee) and were in compliance with the NIH ethical guidelines for the Care and Use of Experimental Animals. All animals were handled for 3 days prior to surgery. On the day of surgery, rats were anesthetized with isoflurane in 100% O_2_ (4% induction, 2% maintenance). Stainless steel guide cannulae (26 ga; Plastics One, Inc) were implanted bilaterally into the prelimbic cortex of the mPFC at a 15° angle to vertical (AP +2.9; ML ± 1.6; DV −3.2 from bregma). Coordinates were based on a rat brain atlas (Paxinos and Watson, 2007). Cannulae were secured to the skull with a stainless steel screw, ethyl cyanoacrylate, and acrylic cement. All animals were given a recovery period of at least 7 days before subsequent behavioral training.

### Behavioral procedures

In all behavioral experiments, rats received immediate post-training bilateral infusions of clasto-Lactacystin β-lactone (β lac; 32 ng/μl; Sigma), anisomycin (ANI; 125 μg/μl; Sigma), or vehicle into the PL mPFC. Both β-lac and ANI were dissolved in 20% DMSO in HCL and diluted in artificial cerebral spinal fluid (aCSF). Control rats were given infusions of 20% DMSO diluted in aCSF. Each infusion was given at a rate of 0.3 μl/min with a total volume of 0.3 μl/side. Concentration of β-lac and ANI as well as total infusion volume were taken from previous work on memory consolidation in the amygdala and PFC (Gilmartin and Helmstetter, 2010; Jarome et al., 2011; Kwapis et al., 2011). The injectors remained in place for an additional 90 s to ensure sufficient diffusion of the drug. After infusion, the obdurators were re-inserted into the cannulae and the animal was returned to its home cage.

All conditioning sessions occurred in a set of four identical Plexiglas and stainless-steel chambers housed inside sound-attenuating boxes. Each outer box was illuminated by a 7.5 watt house light and contained a ventilation fan with a background noise level of 62–64 dB. The floors of the Plexiglas chambers (Context A) were made of evenly spaced stainless steel rods through which the foot-shock was delivered. Additionally, each chamber was cleaned and wiped down with 5% ammonium hydroxide between each set of rats.

After the 7 day recovery period, all animals received 3 days of transport and handling in which they were habituated to the infusion procedure. During transport handling, each rat was lightly restrained in a towel and the infusion pump was activated to habituate the animal to the noise. On the day of training, rats were placed into the conditioning chambers and were given a 6 min baseline period followed by either 4 trials of DFC or 6 trials of trace fear conditioning. These protocols typically result in similar conditional responding to the CS (Kwapis et al., 2011). Each DFC trial consisted of a 10-s, 72-dB white noise CS and a 1-s, 1-mA foot-shock UCS. Each trial of DFC was separated by an inter-trial interval (ITI) of 110 ± 20 s. Each trace fear conditioning (TFC) trial consisted of the same CS and UCS separated by a stimulus-free 20-s trace interval (ITI 240 ± 20 s). To analyze the percent freezing during training, the training session was divided into 3 distinct phases. The baseline phase represents the first 6 min of training wherein neither stimulus is presented. This is followed by the CS-UCS phase in which the CS-UCS pairings are given. The last 3 min of training represent a post training phase after which the animal was removed from the training context and returned to its homecage. Immediately after the training session, each rat was injected with ANI, β-LAC, or vehicle. In this experiment, there were 6 total groups (TFC β-lac, *n* = 11; TFC ANI, *n* = 10; TFC VEH, *n* = 9; DFC β-lac, *n* = 10; DFC ANI, *n* = 7; DFC VEH, *n* = 10).

Approximately 24 h after training, each rat was tested for fear to the auditory CS in a novel context (Context B). Context B was illuminated with an infrared light and had opaque white floor panels. Before testing each rat, the walls of the context B were wiped with 5% acetic acid solution. After a 1-min baseline, rats were given 8, 30-s presentations of the CS (ITI 60 s). Rats were removed from the chamber immediately following the final CS presentation. To test for context memory, rats were placed back into the training context (Context A) for 12 min with no CS or US presentations. The percent of time spent freezing during the entire period was used as the dependent measure. The CS test and context test were counterbalanced and occurred 4 h apart. Fear to the auditory CS and to the training context were tested a second time, 48 h after training, using the same test procedures.

After testing, animals were overdosed with isoflurane and transcardially perfused with saline followed by 10% buffered formalin. The heads were placed in formalin for 24 h. The brains were then removed from the skull and cryo-protected in 20% sucrose formalin. Each brain was then sectioned through the prelimbic region of the PFC (40 μm). The sections were mounted on slides and stained with cresyl violet. The infusion sites were then verified using a rat brain atlas (Paxinos and Watson, 2007).

The behavior of each rat during training and testing was recorded on digital video. The percent time freezing was determined through frame-by-frame analysis of pixel changes using FreezeScan 2.0 software (Clever Sys, Inc.). The automatic scoring parameters were chosen to match hand-scoring parameters previously used in our laboratory to measure freezing.

### Western immunoblotting

For western blot experiments, rats were trained using the same delay (*n* = 11) and trace (*n* = 9) fear conditioning procedures described above but were sacrificed 60 min following the training session. Home cage control (HC; *n* = 10) animals were sacrificed throughout the day. Brains were immediately removed and placed on dry ice and then stored at −80°C until dissected. Prefrontal cortical tissue was dissected out, homogenized in buffer (in 100 ml DDH_2_O;.605 g Tris Base, 0.25 g sodium deoxycholate, 0.876 g NaCl,.038 g EDTA, 0.0042 g sodium fluoride, 1 μg/ml PMSF, 1 μg/ml aprotinin, 1 μg/ml leupeptin, 10 ml 10% SDS, 1 mM sodium orthovanadate), and stored at −80°C. The samples were thawed and centrifuged at 4000 rpm for 20 min. A Bradford protein assay kit (BioRad) was then used to measure protein concentration.

Each sample was then loaded into a 7.5% SDS-PAGE gel. The separated proteins were transferred onto PVDF membranes using a Turbo Transfer system (BioRad). Membranes were incubated in blocking buffer for 1 h before being incubated at 4°C overnight in primary antibody for K48 polyubiquitin (Millipore) and actin (Cell Signaling). The next day, the membranes were incubated in secondary antibody (dilution 1:30,000; Upstate Biotechnology anti-rabbit) for 1 h. Membranes were then washed and soaked in a chemiluminescence solution for 5 min (Supersignal West Dura, Thermo).

Images were captured using the G-BOX Chemi XT-4 camera system (Syngene). The mean optical density for each sample was analyzed with GeneSYS analysis software (Syngene). The optical density of K48 polyubiquitination for each sample was normalized to the optical density of the loading control, actin, for each sample. A percentage of home cage control value was then derived for each animal by dividing the percent optical density of K48 relative to actin by the percent K48 optical density relative to actin of the home cage. Values were then analyzed with SPSS, using a One-Way ANOVA and Fisher's least significant difference (LSD) *post-hoc* tests.

## Results

**Figure 1:**
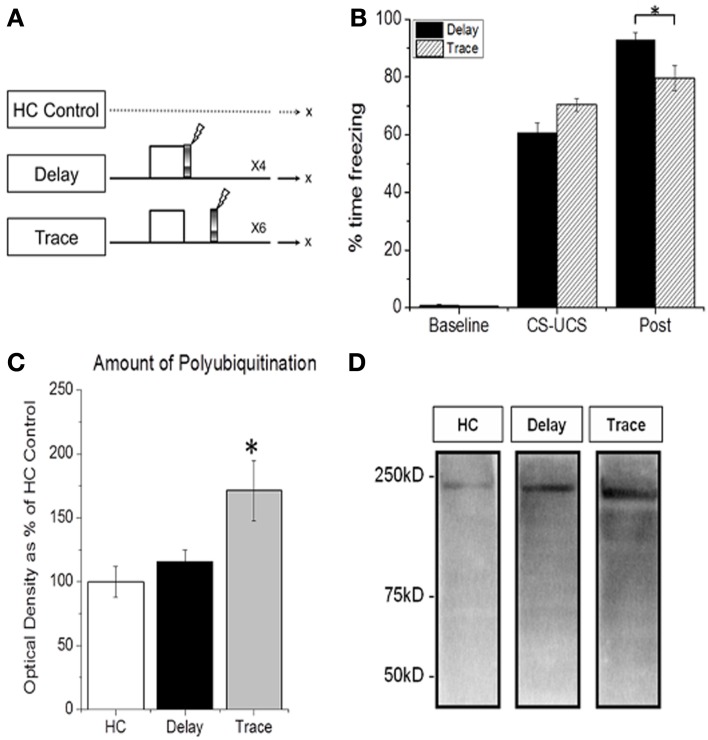
**TFC-specific increase in prefrontal protein degradation. (A)** Training procedure for **(B–D)**. Animals were trained with delay or trace fear conditioning and PFC tissue was collected 60 min later. Home cage (HC) control animals were not trained and PFC tissue was collected throughout the day. **(B)** All animals show normal acquisition of fear conditioning. **(C,D)** Degradation specific polyubiquitination is increased in PFC 60 min after TFC (*n* = 9) but not DFC (*n* = 11), relative to HC animals (*n* = 10). This further supports a selective role for the PFC in trace learning. *Indicates *p* < 0.05 from HC controls.

We first determined whether UPS-mediated protein degradation was up-regulated in the PFC as a result of training with delay vs. trace fear conditioning (Figure 1A). Western blot analysis revealed an increase in degradation specific polyubiquitinated proteins in PFC following trace but not DFC (Figures 1C,D). ANOVA revealed a significant main effect of training [*F*_(2, 32)_ = 4.124, *p* = 0.008]. Fisher LSD *post-hoc* analysis showed that the TFC group was significantly different from home cage controls (*p* = 0.009). The TFC group trended toward a significant increase compared with DFC (*p* = 0.066). Importantly, a mixed model ANOVA revealed no effect of training group [*F*_(1, 19)_ = 0.408, *p* = 0.531], a significant effect of phase within the session [*F*_(1, 19)_ = 928.684, *p* = 0.001] and a significant phase by group interaction [*F*_(1, 19)_ =6.860, *p* = 0.017] on the acquisition of freezing (Figure 1B). A subsequent student's *t*-test confirmed a significant difference in post CS-UCS freezing between DFC and TFC animals [*t*_(19)_ = 2.746, *p* = 0.013] in which animals trained with TFC showed less freezing in the post training phase than those trained with DFC.

**Figure 2:**
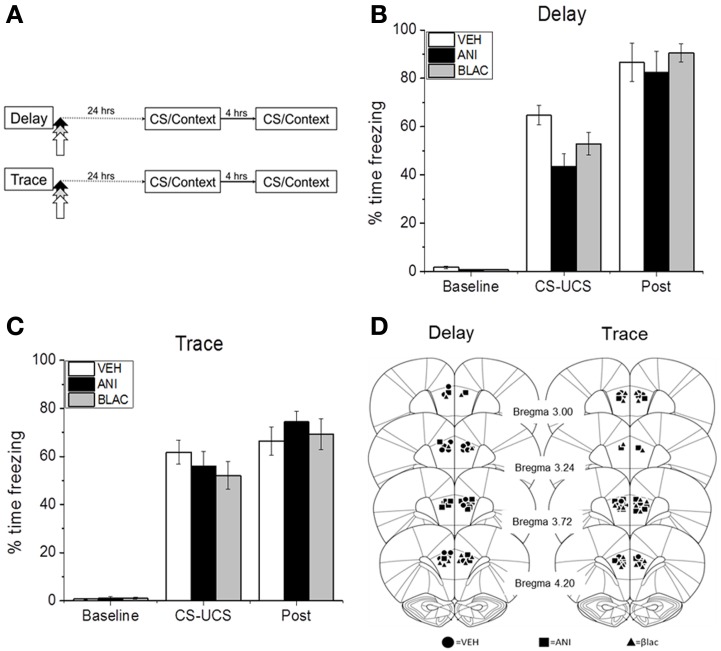
**Acquisition of DFC or TFC. (A)** Training procedure for **(B–D)**. Animals were trained with DFC or TFC and infused with VEH, ANI, or β-Lac immediately after training. **(B)** Mean (±s.e.m) percent time freezing for DFC or TFC **(C)** animals during the baseline period (baseline), the CS-US pairing period (CS-US), and the post-shock period (post) of initial training. **(D)** Locations of injector tips in PL PFC for each group (adapted with permission from Paxinos and Watson, 2007). *p < 0.05.

Next we tested whether the observed increase in degradation after training is necessary for memory consolidation. This experiment also tested whether protein synthesis in PFC is necessary for the consolidation of memory at 24 h. Immediately following trace or delay conditioning rats were injected with inhibitors of protein synthesis or degradation or vehicle (Figure 2A). Rats were tested for memory the following day. All animals showed increased freezing as a result of CS-UCS pairings (Figures 2B,C). Although injections occurred after the training sessions, DFC rats assigned to the ANI and BLAC groups exhibited slightly less freezing during the session. A mixed model ANOVA revealed that this decreased freezing was not statistically reliable as there was no effect of group [*F*_(2, 22)_ = 1.796, *p* = 0.189] and a non-significant group by phase interaction [*F*_(2, 22)_ = 60.677, *p* = 0.287]. As expected, there was a significant main effect of phase with all rats freezing more after training compared to pre-shock baseline [*F*_(2, 22)_ = 423.907, *p* = 0.001]. Immediately after training, rats were either infused with β-lac, the protein synthesis inhibitor ANI, or vehicle. Figure 2D shows location of injector tip for each animal included in the analysis.

**Figure 3:**
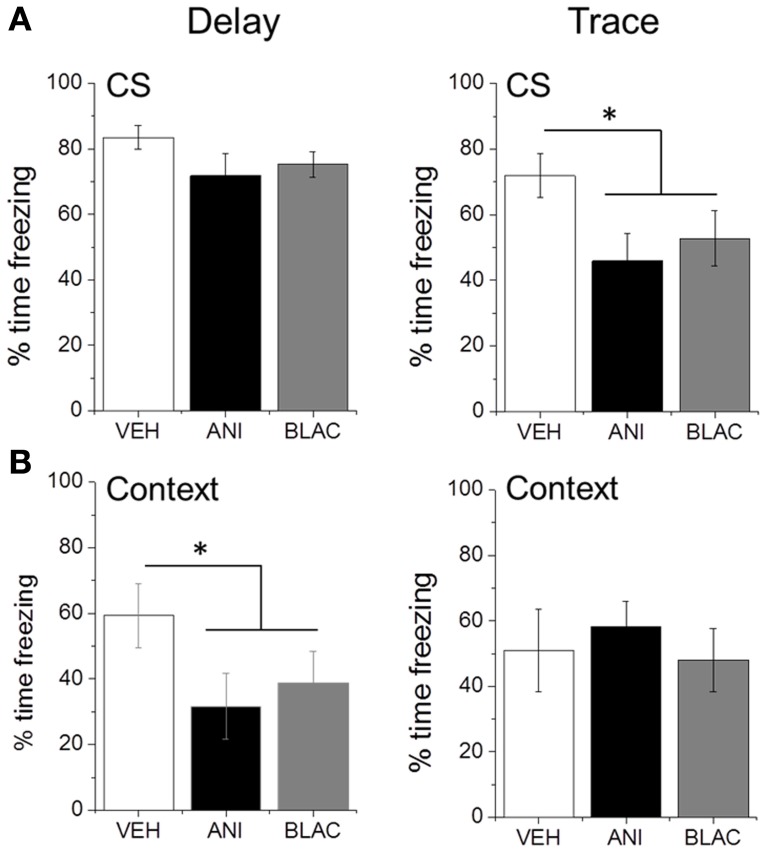
**Consolidation of trace fear memory is impaired by proteasome and protein synthesis inhibition. (A)** Mean percent time freezing during CS presentations for DFC or TFC trained animals. Animals infused with ANI or β-lac and trained with TFC showed a significant reduction in freezing compared to VEH infused animals. Drug infusion did not significantly affect freezing in animals trained with DFC. **(B)** Mean percent time freezing during context test for DFC or TFC trained animals. Infusions of ANI or β-lac significantly reduced context freezing in DFC animals but not TFC animals. *Indicated *p* < 0.05 from VEH.

Rats were tested for fear to the auditory CS and training context the next day. Blocking protein degradation or protein synthesis in PFC immediately after trace, but not delay training, impaired memory for the CS. ANOVA revealed a trend for a main effect of Group for animals trained with TFC [*F*_(2, 27)_ = 2.785, *p* = 0.079] but not with DFC [*F*_(2, 24)_ = 1.535, *p* = 0.236]. *Post-hoc* analysis using Fisher's Least Significant Difference test revealed no significant difference in CS freezing (Figure 3A) between the drug-treated groups trained with TFC (*p* = 0.461) or between the drug-treated groups trained with DFC (*p* = 0.968). Therefore, the drug-treated groups were collapsed and a planned comparison between the collapsed drug groups and the vehicle group revealed a significant reduction in CS freezing for drug-treated animals trained with TFC (*p* = 0.032) but not DFC (*p* = 0.094) compared to VEH animals trained with TFC or DFC, respectively. Prefrontal protein synthesis or degradation was not necessary for contextual fear memory in TFC animals [*F*_(2, 27)_ = 0.117, *p* = 0.890]. Blocking protein synthesis or degradation did impair background contextual fear conditioning in DFC trained animals with a non-significant trend toward reduced contextual fear [*F*_(2, 24)_ = 2.773, *p* = 0.083]. Again, *post-hoc* analysis revealed no differences in context freezing between DFC animals infused with ANI or β-lac (*p* = 0.674), so the drug-treated groups were collapsed. Planned comparisons revealed significantly lower context freezing in the drug-treated groups compared to the vehicle infused group for animals trained with delay (*p* = 0.027) but not trace (*p* = 0.849) fear conditioning (Figure 3B).

## Discussion

The present study may be the first to demonstrate the critical involvement of ubiquitin-proteasome mediated protein degradation in the consolidation of a memory that depends specifically on the PFC. We found an increase in degradation specific polyubiquitination in the PFC following trace but not DFC. We further demonstrate a functional role for prefrontal UPS-mediated degradation in the consolidation of memory. Inhibiting the proteolytic activity of the UPS in the PFC immediately after trace fear conditioning impairs auditory CS memory when tested the next day. In addition to protein degradation, *de novo* protein synthesis in PFC is also necessary for memory consolidation. Together, our results suggest that both protein degradation via the UPS and *de novo* protein synthesis are critical for the initial consolidation of trace fear memory involving cells in the PFC.

The contribution of UPS-mediated proteolysis to learning and memory is gaining increasing support. In aplysia, the degradation of specific inhibitory proteins results in a facilitation of a signaling cascade involved in transcription and translation, ultimately leading to the consolidation of long-term facilitation (Hegde et al., 1997). In mammals, proteasome inhibition in the CA1 region of hippocampus resulted in a complete impairment in memory for a one-trial inhibitory avoidance task (Lopez-Salon et al., 2001). However, few studies have examined the role of the UPS in auditory fear memory consolidation and no studies thus far have examined its role in the consolidation of auditory trace fear memory. Here, we provide additional support for the PFC as a site of synaptic plasticity in TFC and further augment the role of the UPS in memory consolidation. Our results indicate that protein degradation in the PFC is critically involved in the initial consolidation of auditory trace fear memories.

While our findings further support the critical involvement of UPS mediated proteolysis in memory consolidation, the proteins targeted for activity-dependent degradation by the UPS remain relatively unknown. Jarome et al. (2011) provided some evidence for the learning related degradation of synaptic scaffolding proteins, such as SHANK, as well as a RNA helicase, known as MOV10, in the amygdala following DFC. The activity-dependent degradation of SHANK and MOV10 is believed to contribute to the destabilization of synapses after memory retrieval which is critical for the subsequent synaptic restabilization. The learning induced degradation of functionally disparate proteins illustrates the multi-faceted role of UPS-mediated protein degradation in learning and memory. Now that we have found that UPS-mediated degradation in PFC supports memory consolidation similarly to its role in amygdala and hippocampus, future work can investigate specific proteins being specifically targeted degradation in this brain structure.

Successful memory consolidation may require a balance between protein degradation and synthesis (Jarome and Helmstetter, 2013). We found that both are necessary in the PFC for trace fear conditioning. Blocking protein synthesis with ANI impaired TFC, consistent with previous work. Dash and colleagues showed that bilateral mPFC infusions of the protein synthesis inhibitor, anisomycin, immediately following TFC impaired memory tested at a remote time point 30 days later (Blum et al., 2006). We show that even recent memory requires protein synthesis. This is an important finding given that post-training lesions may not impair TFC (Quinn et al., 2008). Animals whose PFC is lesioned 2 days after training exhibit intact freezing at subsequent testing, suggesting PFC is not a site of permanent storage of TFC memory. It is likely that storage of this memory is distributed, but our results and those of Dash clearly demonstrate that the consolidation of memory requires protein synthesis and degradation following training.

Given previous work from our lab showing that contextual fear in both trace and delay conditioning are similarly affected by manipulation of prefrontal activity (Gilmartin and Helmstetter, 2010), it is somewhat surprising that we saw different patterns of context freezing between trace and delay conditioning in the present study. Our data show a significant effect of proteasome or protein synthesis inhibition on context freezing for animals trained with delay but not trace fear conditioning. However, one possible explanation for this discrepancy is that animals trained in DFCrecieved fewer footshocks (4) in the training context than animals trained in TFC (6).Furthermore, rats were tested for “background” context memory (i.e., the auditory CS was present during training). Together, this could make it difficult to make a conclusion about the impairment in contextual fear memory of DFC animals. Nevertheless, additional studies may be required to resolve this issue.

Protein synthesis is generally accepted as a mechanism of synaptic plasticity that is necessary for fear memory consolidation (Schafe and LeDoux, 2000; Maren et al., 2003; Parsons et al., 2006; Helmstetter et al., 2008). Additionally, UPS- mediated degradation has been shown to occur in parallel with protein synthesis in the amygdala to support memory consolidation following DFC (Jarome et al., 2011). We have demonstrated that both *de novo* protein synthesis and protein degradation in the PFC are necessary and critical to the formation of trace fear memories. The concurrence of these two mechanisms suggests that they may act in concert and make up a larger regulatory mechanism of synaptic plasticity. There is some evidence that suggests that the UPS plays a role in regulating mechanisms involved in transcription or translation (Ehlers, 2003; Ghosh et al., 2008; Banarjee et al., 2009). Given the involvement of both mechanisms in memory formation, the idea of a reciprocal relationship between protein synthesis and protein degradation will certainly be of great interest in future studies.

While the specific protein-protein interactions may vary based on the learning paradigm, both protein degradation and *de novo* protein synthesis, in several brain structures, are critical for several types of learning. Our findings, taken together with previous work, may suggest the existence of a generalized and perhaps more unified mechanism of plasticity; one in which UPS-mediated proteolysis and protein synthesis function in a reciprocal fashion. Additional research should address the functional relationship between *de novo* protein synthesis and UPS-mediated proteolysis.

### Conflict of interest statement

The authors declare that the research was conducted in the absence of any commercial or financial relationships that could be construed as a potential conflict of interest.
